# The prevalence of injection-site reactions with disease-modifying therapies and their effect on adherence in patients with multiple sclerosis: an observational study

**DOI:** 10.1186/1471-2377-11-144

**Published:** 2011-11-10

**Authors:** Karsten Beer, Martin Müller, Anna Marie Hew-Winzeler, Adriano Bont, Philippe Maire, Xiaojun You, Pamela Foulds, Jessica Mårlind, Daniela Curtius

**Affiliations:** 1 Private practice, Obere Bahnhofstrasse, CH 9500, Wil, Switzerland; 2 Department of Medicine, Canton Hospital of Lucerne, Lucerne, Switzerland; 3 Private practice, Todistrasse 46, CH 8002, Zurich, Switzerland; 4 Private practice, Brunngasse 6, CH 8400, Winterthur, Switzerland; 5 Hirslanden Clinic Aarau, Schanisweg, CH 5001, Aarau, Switzerland; 6 Biogen Idec Inc., 133 Boston Post Road, Weston, MA, USA; 7 Biogen-Dompé AG, Bundesplatz 9, CH 6300, Zug, Switzerland

## Abstract

**Background:**

Interferon beta (IFNβ) and glatiramer acetate (GA) are administered by subcutaneous (SC) or intramuscular (IM) injection. Patients with multiple sclerosis (MS) often report injection-site reactions (ISRs) as a reason for noncompliance or switching therapies. The aim of this study was to compare the proportion of patients on different formulations of IFNβ or GA who experienced ISRs and who switched or discontinued therapy because of ISRs.

**Methods:**

The Swiss MS Skin Project was an observational multicenter study. Patients with MS or clinically isolated syndrome who were on the same therapy for at least 2 years were enrolled. A skin examination was conducted at the first study visit and 1 year later.

**Results:**

The 412 patients enrolled were on 1 of 4 disease-modifying therapies for at least 2 years: IM IFNβ-1a (n = 82), SC IFNβ-1b (n = 123), SC IFNβ-1a (n = 184), or SC GA (n = 23). At first evaluation, ISRs were reported by fewer patients on IM IFNβ-1a (13.4%) than on SC IFNβ-1b (57.7%; *P *< 0.0001), SC IFNβ-1a (67.9%; *P *< 0.0001), or SC GA (30.4%; *P *= not significant [NS]). No patient on IM IFNβ-1a missed a dose in the previous 4 weeks because of ISRs, compared with 5.7% of patients on SC IFNβ-1b (*P *= 0.044), 7.1% of patients on SC IFNβ-1a (*P *= 0.011), and 4.3% of patients on SC GA (*P *= NS). Primary reasons for discontinuing or switching therapy were ISRs or lack of efficacy. Similar patterns were observed at 1 year.

**Conclusions:**

Patients on IM IFNβ-1a had fewer ISRs and were less likely to switch therapies than patients on other therapies. This study may have implications in selecting initial therapy or, for patients considering switching or discontinuing therapy because of ISRs, selecting an alternative option.

## Background

Interferon beta (IFNβ) and glatiramer acetate (GA) are first-line therapies for the long-term treatment of multiple sclerosis (MS) and are generally believed to have comparable efficacy. IFNβ-1b, IFNβ-1a, and GA are administered by either intramuscular (IM) or subcutaneous (SC) injection, the frequency of which varies from daily to weekly depending on the product. The injection of these therapies can be associated with severe adverse skin reactions, such as necrosis and lipoatrophy [1-4], which patients may experience after years of treatment. The frequency of injections and injection-site reactions (ISRs) can be burdensome for patients and these reactions are, among other side effects, a major reason for poor adherence with these medications over the long term [5-7]. O'Rourke and Hutchinson reported that patients stopped IFNβ therapy because of side effects after a median of 13 months [6]. Since poor medication adherence or discontinuation can lead to treatment failure and ultimately poorer long-term prognosis, it is critical that patients persist with treatment to achieve maximum benefit from therapy. The objective of this observational study was to compare the proportion of patients on different formulations of IFNβ or GA who experienced ISRs and who switched or discontinued therapy because of ISRs.

## Methods

### Study design and participants

The Swiss MS Skin Project was an observational study supported by Biogen Idec Inc. and conducted at 31 sites in Switzerland. This was a noninterventional and observational study; therefore, approval from an ethics committee or health authority was not required. Written informed consent was obtained from all patients before any study-related procedures were performed.

Men or women between the ages of 18 and 55 years with relapsing MS or clinically isolated syndrome (CIS) were enrolled. Information on demographic characteristics, current MS therapy and duration, and concomitant disease and medication were collected at study entry.

Patients were not naive to therapy and were required to be on 1 of 4 disease-modifying therapies (DMTs) for at least 2 years prior to enrollment: IM IFNβ-1a (Avonex^®^), SC IFNβ-1b (Betaseron^®^/Betaferon^®^), SC IFNβ-1a (Rebif^®^), or SC GA (Copaxone^®^). Rebif New Formulation was not approved in Switzerland at the time of this study. A thorough skin exam was performed on patients who met all inclusion criteria. During this first evaluation, all observed ISRs were recorded, with additional data (number of occurrences and severity) collected for cases of necrosis and lipoatrophy. Patients were asked whether an injection was omitted in the previous 4 weeks because of skin reactions, and, if so, the number of injections missed was recorded. Changes in therapy, including treatment discontinuation, or a patient's desire to change or discontinue treatment and the reason were also noted. A follow-up skin evaluation specific for necrosis and lipoatrophy was performed 1 year later or at early termination from the study. At the follow-up evaluation, changes in treatment and the reason for the change were recorded.

The primary outcomes of this study were to evaluate the proportion of patients on IFNβ or GA who experienced ISRs and who switched or discontinued therapy because of ISRs.

### Statistical methods

A chi-square test and a Fisher exact test were used for comparisons between categorical variables (gender, ISR, necrosis, lipoatrophy, injection omitted, and treatment changed). *t *tests were used for comparisons between continuous variables (age, duration of disease, and treatment duration).

## Results

### Patient disposition and disease characteristics

Of the 501 patients screened at the start of the study, 412 met per-protocol entry criteria. Patients were currently on 1 of 4 DMTs: IM IFNβ-1a (n = 82), SC IFNβ-1b (n = 123), SC IFNβ-1a (n = 184), or SC GA (n = 23). There were no significant differences in any study entry demographic or disease characteristic across treatment groups (Table 1). The overall mean age at enrollment was 44.3 years (standard deviation [SD] 10.12) and the majority of patients (69.7%) were women. The overall mean duration of MS disease was 9.3 years (SD 6.22) and the overall mean duration of treatment was 5.9 years (SD 2.97). Overall, 88 of 412 patients (21.4%) were taking comedication for a concomitant disease.

**Table 1:** Demographic and disease characteristics of patients^a ^at study entry

	IM IFNβ-1a (n = 82)	SC IFNβ-1b (n = 123)	SC IFNβ-1a (n = 184)	SC GA (n = 23)	Overall (N = 412)
Mean age, years (SD)	44.7 (10.35)	45.9 (10.23)	43.2 (9.72)	43.1 (11.25)	44.3 (10.12)
Gender, female (%)	64 (78.0)	84 (69.4)	122 (66.7)	15 (65.2)	285 (69.7)
Mean duration of MS disease, years (SD)	9.9 (7.37)	10.0 (6.29)	8.6 (5.74)	9.5 (4.92)	9.3 (6.22)
Mean duration of treatment, years (SD)	5.5 (2.56)	6.8 (3.06)	5.5 (2.93)	5.6 (3.24)	5.9 (2.97)

^a^There were no statistically significant differences between the groups.

The end-of-study visit was completed for 351 patients: 73 patients on IM IFNβ-1a, 107 patients on SC IFNβ-1b, 156 patients on SC IFNβ-1a, and 15 patients on SC GA. The reasons for study discontinuation included lost to follow-up (n = 37), stopped therapy (n = 8), changed physicians (n = 6), switched therapy to natalizumab (n = 3) or mitoxantrone (n = 1), withdrew consent (n = 3), pregnancy (n = 1), deterioration of general condition (n = 1), and protocol violation (inclusion criteria not met, n = 1).

### First evaluation

**Figure 1:**
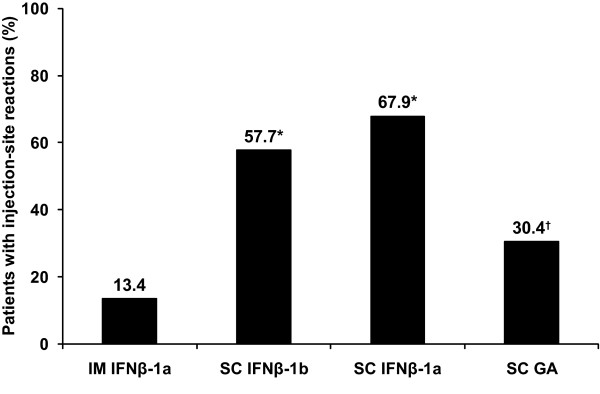
**Results of skin examination at the first evaluation**. The number of patients experiencing ISRs at the first evaluation was recorded for each therapy. At the first evaluation examination, patients had been on their current DMT for at least 2 years. *P < 0.0001 vs IM IFNβ-1a; chi-square test. ^†^*P *= 0.016 vs SC IFNβ-1b; *P *< 0.001 vs SC IFNβ-1a; *P *= NS vs IM IFNβ-1a; chi-square test.

Of the 412 patients enrolled in this study, 214 (51.9%) reported an adverse skin reaction at the first evaluation. ISRs were reported for fewer patients on IM IFNβ-1a (11 of 82 patients, 13.4%) than on SC IFNβ-1b (71 of 123 patients, 57.7%; *P *< 0.0001), SC IFNβ-1a (125 of 184 patients, 67.9%; *P *< 0.0001), or SC GA (7 of 23 patients, 30.4%; *P *= not significant [NS]) (Figure 1). The proportion of SC GA-treated patients who experienced ISRs was also significantly lower than that for SC IFNβ-1b (*P *= 0.016) and SC IFNβ-1a (*P *< 0.001). Of the 214 patients who experienced an ISR, 123 (57.5%) reported that ISRs occurred "several times," 74 (34.6%) reported that they occurred "frequently," and 8 (3.7%) reported that they occurred "once." Data were not available for 9 patients (4.2%).

Necrosis was reported for 18 of 412 patients (4.4%) at the first evaluation (Table 2). No patients on IM IFNβ-1a or SC GA experienced necrosis, compared with 7 of 123 patients (5.7%) treated with SC IFNβ-1b and 11 of 184 patients (6.0%) treated with SC IFNβ-1a. Most of the patients who experienced necrosis (77.8%) had 1-2 occurrences. The severity of necrosis was rated as mild or moderate in the majority (83.3%) of these patients; severe necrosis was reported in 1 of 7 patients (14.3%) treated with SC IFNβ-1b and 1 of 11 patients (9.1%) treated with SC IFNβ-1a. Data on the frequency and severity of necrosis were unknown for 1 patient receiving SC IFNβ-1a.

**Table 2:** Skin status at first evaluation examination

	IM IFNβ-1a (n = 82)	SC IFNβ-1b (n = 123)	SC IFNβ-1a (n = 184)	SC GA (n = 23)	Overall (N = 412)
Any ISR, n (%)	11 (13.4)	71 (57.7)	125 (67.9)	7/23 (30.4)	214 (51.9)
Necrosis, n (%)	0 (0)^a^	7 (5.7)	11 (6.0)	0 (0)	18 (4.4)
Number of occurrences					
1	0	1/7 (14.3)	6/11 (54.5)	0	7/18 (38.9)
2	0	4/7 (57.1)	3/11 (27.3)	0	7/18 (38.9)
3	0	1/7 (14.3)	1/11 (9.1)	0	2/18 (11.1)
4	0	1/7 (14.3)	0	0	1/18 (5.6)
Unknown	0	0	1/11 (9.1)	0	1/18 (5.6)
Severity					
Mild	0	3/7 (42.9)	6/11 (54.5)	0	9/18 (50.0)
Moderate	0	3/7 (42.9)	3/11 (27.3)	0	6/18 (33.3)
Severe	0	1/7 (14.3)	1/11 (9.1)	0	2/18 (11.1)
Unknown	0	0	1/11 (9.1)	0	1/18 (5.6)
Lipoatrophy, n (%)	1 (1.2)^b^	11 (8.9)	19 (10.3)	3 (13.0)	34 (8.3)
Number of occurrences					
1	0	1/11 (9.1)	5/19 (26.3)	1/3 (33.3)	7/34 (20.6)
2	0	6/11 (54.5)	6/19 (31.6)	1/3 (33.3)	13/34 (38.2)
3	0	2/11 (18.2)	2/19 (10.5)	1/3 (33.3)	5/34 (14.7)
4	1/1 (100)	0	0	0	1/34 (2.9)
≥5	0	2/11 (18.2)	6/19 (31.6)	0	8/34 (23.5)
Severity					
Mild	1/1 (100)	6/11 (54.5)	10/19 (52.6)	2/3 (66.7)	19.34 (55.9)
Moderate	0	2/11 (18.2)	9/19 (47.4)	1/3 (33.3)	12/34 (35.3)
Severe	0	1/11 (9.1)	0	0	1/34 (2.9)
Unknown	0	2/11 (18.2)	0	0	2/34 (5.9)
Injection omitted during last 4 weeks, n (%)	0 (0)^c^	7 (5.7)	13 (7.1)	1 (4.3)	21 (5.1)

At the first evaluation examination, patients had been on their current DMT for at least 2 years.

^a^*P *= 0.028 vs SC IFNβ-1b; *P *= 0.020 vs SC IFNβ-1a.

^b^*P *= 0.021 vs SC IFNβ-1b; *P *= 0.009 vs SC IFNβ-1a; *P *= 0.032 vs SC GA.

^c^*P *= 0.044 vs SC IFNβ-1b; *P *= NS vs SC GA; *P *= 0.011 vs SC IFNβ-1a.

At the first evaluation, lipoatrophy was reported for 34 of 412 patients (8.3%) (Table 2): 1 of 82 patients (1.2%) treated with IM IFNβ-1a, 11 of 123 patients (8.9%) treated with SC IFNβ-1b, 19 of 184 patients (10.3%) treated with SC IFNβ-1a, and 3 of 23 patients (13.0%) treated with SC GA. Lipoatrophy was experienced by significantly fewer patients on IM IFNβ-1a than on SC IFNβ-1b (*P *= 0.021), on SC IFNβ-1a (*P *= 0.009), or on SC GA (*P *= 0.032). The majority of patients who experienced lipoatrophy (73.5%) had 1-3 occurrences. Five or more occurrences of lipoatrophy were seen in 2 of 11 patients (18.2%) treated with SC IFNβ-1b and 6 of 19 patients (31.6%) treated with SC IFNβ-1a. The severity of lipoatrophy was rated as mild or moderate in most patients (91.2%); severe lipoatrophy was reported for 1 of 11 patients (9.1%) treated with SC IFNβ-1b. Data on severity were unknown for 2 patients receiving SC IFNβ-1b.

**Figure 2:**
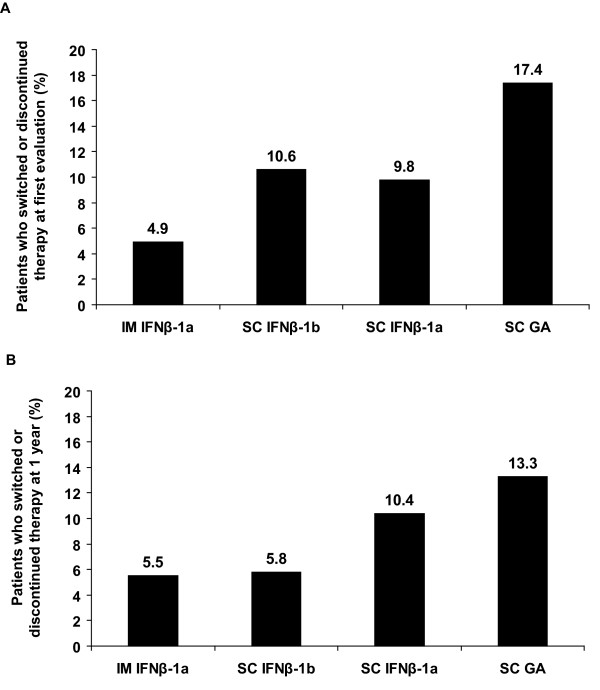
**Patients wanting to switch or discontinue therapy at first evaluation (A) and 1 year (B)**. At the first evaluation examination, patients had been on their current DMT for at least 2 years.

**Figure 3:**
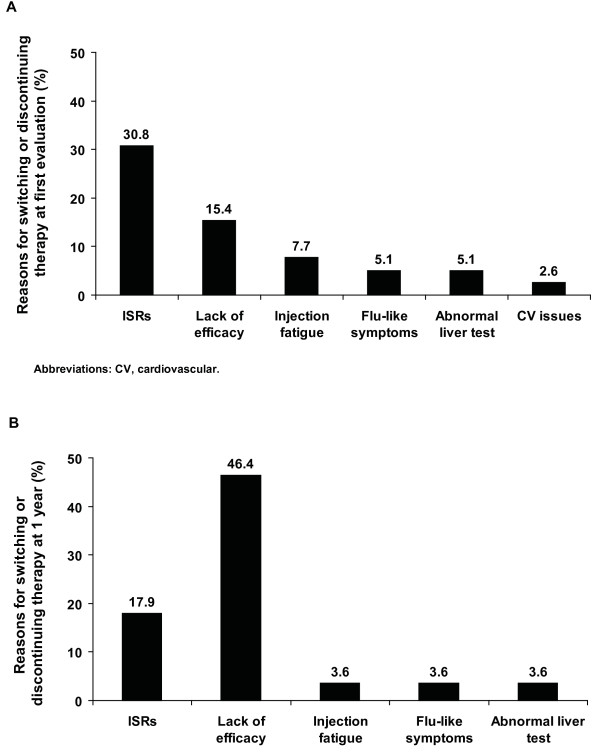
**Reasons for switching or discontinuing therapy at the first evaluation (A) and 1 year (B)**. At the first evaluation examination, patients had been on their current DMT for at least 2 years.

At the first evaluation, no patient on IM IFNβ-1a had missed a dose in the previous 4 weeks, compared with 7 of 123 patients (5.7%) treated with SC IFNβ-1b (*P *= 0.044), 13 of 184 patients (7.1%) treated with SC IFNβ-1a (*P *= 0.011), and 1 of 23 patients (4.3%) treated with SC GA (*P *= NS) (Table 2). Overall, 39 of 412 patients (9.5%) reported wanting to switch therapies or discontinue therapy at the first evaluation: 4 of 82 patients (4.9%) treated with IM IFNβ-1a, 13 of 123 patients (10.6%) treated with SC IFNβ-1b, 18 of 184 patients (9.8%) treated with SC IFNβ-1a, and 4 of 23 patients (17.4%) treated with SC GA (Figure 2A). The primary reasons for wanting to switch therapy were ISRs (30.8%) and lack of efficacy (15.4%). Other reasons included injection fatigue (7.7%), flu-like symptoms (5.1%), abnormal liver function (5.1%), and cardiovascular problems (2.6%) (Figure 3A). One patient on IM IFNβ-1a switched therapy because of ISRs or injection fatigue, compared with 7 patients on SC IFNβ-1b and 7 patients on SC IFNβ-1a. No patients on SC GA switched therapy because of an ISR or injection fatigue.

### Follow-up skin evaluation at 1 year

**Figure 4:**
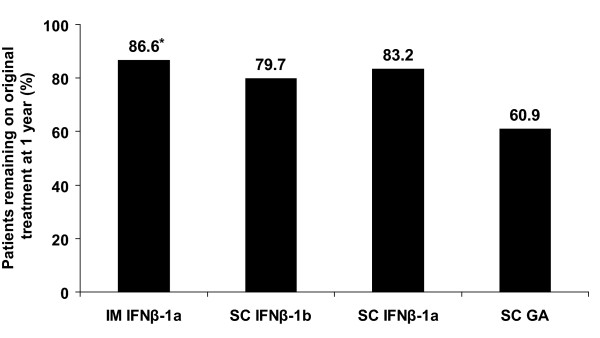
**Patients remaining on original treatment at 1-year follow-up**. *Overall *P *= 0.036 (chi-square test).

Adherence was comparable among the 4 DMTs at 1 year. However, a significantly higher proportion of patients who started on IM IFNβ-1a were still on the same therapy (86.6%) compared with patients on SC IFNβ-1b (79.7%), patients on SC IFNβ-1a (83.2%), and patients on SC GA (60.9%) (overall *P *= 0.036 [chi-square test]) (Figure 4).

Data on necrosis were collected from 345 patients. Ten of 345 patients (2.9%) experienced necrosis at the injection site: 5 of 104 patients (4.8%) treated with SC IFNβ-1b and 5 of 154 patients (3.2%) treated with SC IFNβ-1a (Table 3). No patients on IM IFNβ-1a or SC GA experienced necrosis. The majority of patients who experienced necrosis (80.0%) reported 1-2 occurrences, and the severity was mild or moderate in most patients (70.0%). Severe necrosis was observed in only 1 patient treated with SC IFNβ-1b. Data on the frequency and severity of necrosis were unknown for 1 patient on SC IFNβ-1b and 1 patient on SC IFNβ-1a.

**Table 3:** Skin status at one-year follow-up examination

	IM IFNβ-1a (n = 73)	SC IFNβ-1b (n = 104)	SC IFNβ-1a (n = 154)	SC GA (n = 14)	Overall (N = 345)
Necrosis, n (%)	0 (0)	5 (4.8)	5 (3.2)	0 (0)	10 (2.9)
Number of occurrences					
1	0	3/5 (60.0)	2/5 (40.0)	0	5/10 (50.0)
2	0	1/5 (20.0)	2/5 (40.0)	0	3/10 (30.0)
Unknown	0	1/5 (20.0)	1/5 (20.0)	0	2/10 (20.0)
Severity					
Mild	0	3/5 (60.0)	1/5 (20.0)	0	4/10 (40.0)
Moderate	0	0	3/5 (60.0)	0	3/10 (30.0)
Severe	0	1/5 (20.0)	0	0	1/10 (10.0)
Unknown	0	1/5 (20.0)	1/5 (20.0)	0	2/10 (20.0)
Lipoatrophy, n (%)	0 (0)^a^	9 (8.7)	23 (15.0)^b^	1 (7.1)	33 (9.6)
Number of occurrences					
1	0	3/9 (33.3)	4/23 (17.4)	0	7/33 (21.2)
2	0	2/9 (22.2)	11/23 (47.8)	1/1 (100)	14/33 (42.4)
3	0	3/9 (33.3)	3/23 (13.0)	0	6/33 (18.2)
4	0	0	1/23 (4.3)	0	1/33 (3.0)
≥5	0	1/9 (11.1)	3/23 (13.0)	0	4/33 (12.1)
Unknown	0	0	1/23 (4.3)	0	1/33 (3.0)
Severity					
Mild	0	6/9 (66.7)	11/23 (47.8)	0	17/33 (51.5)
Moderate	0	2/9 (22.2)	6/23 (26.1)	1/1 (100)	9/33 (27.3)
Severe	0	1/9 (11.1)	1/23 (4.3)	0	2/33 (6.1)
Unknown	0	0	5/23 (21.7)	0	5/33 (15.2)

^a^*P *= 0.011 vs SC IFNβ-1b; *P *< 0.0001 vs SC IFNβ-1a.

^b^Lipoatrophy status was not collected for 1 SC IFNβ-1a patient (n = 153).

Data on lipoatrophy were collected from 344 patients. Lipoatrophy was reported in 33 of 344 patients (9.6%): 9 of 104 patients (8.7%) treated with SC IFNβ-1b, 23 of 153 patients (15.0%) treated with SC IFNβ-1a, and 1 of 14 patients (7.1%) treated with SC GA (Table 3). No patient on IM IFNβ-1a experienced lipoatrophy, which was significantly lower than the rate of lipoatrophy on SC IFNβ-1b (*P *= 0.011) and SC IFNβ-1a (*P *< 0.0001). At least 5 occurrences of lipoatrophy were observed in 1 of 9 patients (11.1%) treated with SC IFNβ-1b and 3 of 23 patients (13.0%) treated with SC IFNβ-1a. The severity of lipoatrophy was rated as mild or moderate in the majority of patients (78.8%); severe lipoatrophy was experienced by 1 patient treated with SC IFNβ-1b and 1 patient treated with SC IFNβ-1a. Frequency data were unknown for 1 patient and severity data were unknown for 5 patients in the SC IFNβ-1a-treated group.

Data on switching or discontinuing therapy were collected from 346 patients. Overall, 28 of 346 patients (8.1%) reported wanting to switch or discontinue therapy at the 1-year follow-up: 4 of 73 patients (5.5%) treated with IM IFNβ-1a, 6 of 104 patients (5.8%) treated with SC IFNβ-1b, 16 of 154 patients (10.4%) treated with SC IFNβ-1a, and 2 of 15 patients (13.3%) treated with SC GA (Figure 2B). The primary reasons for wanting to switch or discontinue therapy were lack of efficacy (46.4%) and ISRs (17.9%) (Figure 3B). Other reasons included injection fatigue (3.6%), flu-like symptoms (3.6%), and abnormal liver function (3.6%). No patients on IM IFNβ-1a switched therapy because of ISRs or injection fatigue during the 1-year study, compared with 1 patient on SC IFNβ-1b, 4 patients on SC IFNβ-1a, and 1 patient on SC GA.

A logistic regression model was used to evaluate potential relationships between stopping or switching initial treatment at the year 1 follow-up and baseline age, ISRs, initial treatment, gender, and disease duration. Significant interactions were found for gender and IM IFNβ-1a versus GA. The odds of stopping or switching initial MS treatment at the year 1 follow-up were 2.876 times higher for female patients than for male patients (*P *= 0.0115). Likewise, the odds of stopping or switching initial treatment at the year 1 follow-up were 4.877 times higher for patients on GA than for patients on IM IFNβ-1a (*P *= 0.0215). No significant interactions were found for the other baseline variables.

## Discussion

The Swiss MS Skin Project study is the first study to evaluate the relative frequency and severity of ISRs associated with the 4 injectable DMTs available for patients with MS. In our large patient population of more than 400 patients, approximately half of the patients experienced ISRs at the first evaluation even though they had been on the same therapy for at least 2 years (mean treatment duration = 5.9 years). This finding suggests that ISRs continue to be a concern for patients with MS even after they have been on therapy for several years. Indeed, ISRs and lack of efficacy were the 2 most common reasons for patients' discontinuing treatment or wanting to switch therapy at the first evaluation and 1-year follow-up.

We report potentially clinically important differences in the proportion of patients experiencing ISRs with injectable DMTs. At the first evaluation examination, significantly fewer patients on IM IFNβ-1a than on other IFN formulations experienced ISRs. A trend toward lower rates of ISRs with IM IFNβ-1a than with SC GA was also reported. Missed doses in the previous 4 weeks appeared to be associated with twice the likelihood of discontinuing or switching therapy for patients on SC IFNβ-1b, SC IFNβ-1a, or SC GA as compared with patients on IM IFNβ-1a. ISRs were the most common reason for switching or discontinuing therapy, followed by lack of efficacy and injection fatigue. At the 1-year follow-up, similar patterns of ISRs and of switching or discontinuing therapy were seen. Patients on IM IFNβ-1a were significantly less likely to have necrosis or lipoatrophy than patients on SC IFNβ-1b or SC IFNβ-1a. More patients remained on IM IFNβ-1a than on the other injectable therapies. Although lack of efficacy was the most common reason for switching or discontinuing therapy, ISRs were also a common reason.

Our findings that ISRs are common and that fewer patients treated with IM IFNβ-1a than the other injectable therapies experience ISRs are consistent with the findings of previous randomized, controlled studies comparing the therapies pairwise. The EVIDENCE trial compared IM IFNβ-1a 30 μg once weekly to SC IFNβ-1a 44 μg three times weekly [1]. In this study, ISRs were the most commonly reported adverse event. ISRs were experienced by significantly fewer patients on IM IFNβ-1a than on SC IFNβ-1a (28% vs 83%, *P *< 0.001) [1]. Similarly, the INCOMIN open-label trial compared IM IFNβ-1a 30 μg once weekly with SC IFNβ-1b 250 μg every other day. In this study, ISRs were reported in 8% of patients treated with IM IFNβ-1a compared with 37% of patients treated with SC IFNβ-1b (*P *< 0.0001) [2]. These data are further supported by Milanese et al, who showed occurrence of local reactions in 8% of IM IFNβ-1a-treated patients versus 33% of SC IFNβ-1b-treated patients [3]. Finally, BEYOND, a study of SC IFNβ-1b (250 μg or 500 μg every other day) versus SC GA (20 mg daily), reported that 48%-58% of patients across treatment groups experienced ISRs [4]. The current study is limited by its observational nature and the low number of patients on SC GA, which may be reflective of low utilization of SC GA at the study sites in Switzerland. Given the overall mean treatment duration of 5.9 years, study patients may have received other treatments prior to the current DMT. This possibility is important, since a patient may have different expectations with the first therapy than with the second or third therapy. In addition, these results may not reflect the prevalence of ISRs at the initiation of treatment or early in the treatment regime. Nevertheless, the results are generally consistent with, and may be more reflective of, clinical practice than results from controlled clinical trials. At both the first evaluation and 1-year follow-up examinations, patients on IM IFNβ-1a had fewer skin reactions and were less likely to switch therapies than patients on other therapies.

Maintaining patients with MS on DMTs can be challenging, particularly in the early stage of treatment, when the benefits of therapy may not be obvious to patients and such patients are still adjusting to their medication. Patients with MS commonly cite the frequency of injections and ISRs as reasons for missing doses or switching or discontinuing therapies [5-8]. Patients discontinuing therapy because of side effects, such as ISRs, often do so early during in the course of therapy [6]. Selecting therapy with a lower risk for ISRs and educating patients on strategies to minimize their occurrence may help patients adhere to treatment, thus improving their chances for optimal treatment of MS over the long term. Although many factors are involved when making treatment decisions, our study may have implications in selecting initial therapy or, for patients considering switching or discontinuing therapy because of ISRs, selecting an alternative option.

## Conclusions

More patients remained on IM IFNβ-1a compared with other DMTs over the 1-year study. Patients on IM IFNβ-1a had fewer skin reactions and better compliance and were less likely to switch to other DMTs compared with patients on other therapies. Minimizing the impact of adverse effects is crucial in helping patients adhere to their treatment regimens and in improving their chances of better health over the longer term.

## Competing interests

MM has received grants from Biogen Idec, Teva Neuroscience, Bayer-Schering, and Merck-Serono. XY and PF are employees of Biogen Idec Inc. JM and DC are employees of Biogen-Dompé AG. KB, AMHW, AB, and PM declare that they have no competing interests.

## Authors' contributions

DC, JM, PF, and XY were responsible for the concept and design of the study; DC and JM were responsible for study coordination; XY was responsible for the data analysis; KB, MM, AMHW, AB, and PM collected the clinical data. All authors had full access to the study data, contributed to the interpretation of data, contributed to and critically reviewed the manuscript during its development, and approved the final version of the manuscript for submission.

## Pre-publication history

The pre-publication history for this paper can be accessed here:

http://www.biomedcentral.com/1471-2377/11/144/prepub
